# Parliament and the Profession

**Published:** 1916-06-10

**Authors:** 


### PARLIAMENT AND THE PROFESSION.

### The Death of Nurse Kehoe.

Replying to Mr. Ginnell, who asked why no inquiry had been held into the shooting of Nurse Kehoe by soldiers while she was attending to the wounded in Dublin, Mr. Tennant stated that a careful inquiry into this case had been held, and all the witnesses who could give information had been examined. The conclusion arrived at was that Nurse Kehoe was shot accidentally during the fighting.

### Hospital Equipment in Mesopotamia.

The offer of the Scottish Women's Hospital to place a unit at the disposal of the Government for service in Mesopotamia was refused. Answering Mr. Pollock, who asked what was the ground of the refusal, Mr. Tennant said that the Government of India had been informed that all necessary hospital equipment and *personnel* would be supplied on demand from Army Services, and that there was no need for the employment of small voluntary hospital units.

### Fees of Examining Medical Officers.

In a question he asked the Under-Secretary for War, Mr. Prothero suggested that the public money spent on the examination of recruits under the group system by civil practitioners had been wasted, since men who had been passed as medically fit for service had now to appear before a military medical board. Mr. Tennant explained, however, that the examination made by civil practitioners was the preliminary examination, and that it was necessary that all men should be examined by a medical board when called up for service, irrespectively of whether they had been examined on attestation by a medical board, or at their own request previous to being called up for service.

### The Supply of Artificial Limbs.

In spite of Ministerial statements to the contrary, the belief still seems to persist that sailors and soldiers who have lost limbs in the defence of their country are dependent upon charity for the supply of the necessary artificial limbs. On May 31, replying to Mr. Barnes, Dr. Macnamara definitely denied that this was the case. He said that all artificial limbs for sailors and soldiers, rendered necessary by injuries received on service, were supplied in the first place, and subsequently repairs carried out or replacement, when necessary, made, at the expense of the Admiralty, War Office, or Greenwich Hospital Funds. He added that no sailors or soldiers were dependent on charity for such appliances.

### Infant Mortality.

Asked by Mr. Anderson what steps the Local Government Board was taking to enforce thorough and comprehensive schemes for child and maternity welfare, Mr. Long said that the number of infant deaths in London and the large towns was lower this year than the average of the five preceding years, and the infantile death-rate for London itself for the first quarter of 1916 was lower than that of the corresponding quarter during each of the last ten years. He added that his Department was taking active steps, and with much success, to induce local authorities to adopt and carry out comprehensive schemes for maternity and child welfare. Such schemes were now in working in nearly all the large towns, and throughout a number of counties.

### Absurd Allegations against Red Cross Nurses.

Two absurd questions making comparison between the conduct of Irish Volunteer nurses and English Red Cross nurses were asked by Mr. Ginnell. Alleging that the Irish nurses rendered assistance to the wounded of both sides without distinction, while the English nurses refused assistance to any wounded but those of their own side, he suggested that the Foreign Secretary should ascertain from each neutral Government to which of these two practices it would be prepared to give adhesion at the next Hague Conference. Sir Edward Grey's reply was: "The statements in this question are, I believe, so far removed from the fact that I do not propose to answer it." Mr. Ginnell addressed to Mr. Tennant an equally ridiculous question with regard to the conduct of English Red Cross nurses during the Dublin insurrection; he also alleged that the English troops fired on the Irish Red Cross nurses and forced them to evacuate their headquarters while under fire. Mr. Tennant was able to assure the House that there was no truth in the allegations made in the question against the military authorities or the troops, and further that members of all Red Cross Societies rendered valuable and impartial assistance to all wounded, whether civilians, troops, or rebels.

### Medical Men and Military Service.

The Military Service Bill as finally passed contains a new clause dealing with the exemption of medical practitioners. This clause was inserted by the House of Lords and approved by the House of Commons on May 24, 163 voting in favour and forty-two against. The new clause, which is Clause 7, is as follows:

> Regulations made under the Second Schedule to the principal Act shall provide for the establishment of professional committees to deal with claims for exemption made by duly qualified medical practitioners; and any application made by such a medical practitioner on any ground, other than that of conscientious objection, for a certificate of exemption shall be referred by the Tribunal to whom it is made to such a committee in accordance with those regulations; and the recommendation of the committee on the application shall be binding on any Tribunal constituted under the principal Act.

In moving that this clause be agreed to, Mr. Long said it was the result of very patient examination by the War Office and representatives of the civil members of the medical profession who were in this country. The War Office had reason to be greatly anxious as to the provision of medical men at the Front without at the same time depleting the supply of medical men for civil work here at home. Several speakers opposed the clause on the ground that there was no reason why medical men should have special Tribunals. Mr. Charles Roberts, replying to the critics, pointed out that the local Tribunal could not really know the medical needs of the district so well as the Central Medical Committee.

### Other Matters of Interest.

Invalid Prisoners of War in Switzerland.—Answering Mr. Malcolm, Sir Edward Grey stated that the question of the inclusion of civilians in the arrangement arrived at between Great Britain and Germany for the internment of invalid prisoners of war not entitled to repatriation was under consideration.

